# Household food insecurity and coping strategies among rural households in Kedida Gamela District, Kembata-Tembaro zone, Southern Ethiopia: mixed-methods concurrent triangulation design

**DOI:** 10.1186/s40795-022-00663-z

**Published:** 2023-01-03

**Authors:** Gizachew Yohannes, Eskinder Wolka, Temesgen Bati, Tadele Yohannes

**Affiliations:** 1 Kembata-Tembaro Zone, Durame town Administration, Durame, Ethiopia; 2 School of Public Health, College of Medicine and Health Sciences, Wolaita Sodo University, Wolaita Sodo, Ethiopia; 3 School of Public Health, College of Medicine and Health Sciences, Wachemo University, Hossana, Ethiopia

**Keywords:** Household food insecurity, Copping strategies, Ethiopia

## Abstract

**Background:**

Household food insecurity is a state in which household members experienced limited or uncertain physical and economic access to safe, plenty, and healthy food to meet the dietary needs for a fruitful, healthy, and active life. Food insecurity continues to be a major development and public health problem across the globe, having adverse consequences. This study was done to assess household food insecurity and to explore coping strategies in Kedida Gamela District, Southern Ethiopia.

**Method:**

A cross-sectional study complemented with the qualitative inquiry was carried-out from August to November 2020. Multistage sampling was used to select **s**tudy subjects. A total sample of 597 households was selected randomly using up to date family folder list in the district as a sampling frame. For the qualitative study, 16 food-insecure households were selected randomly from food in secured households. Quantitative data were entered using Ep-Data 3.1 and exported to SPSS 20 for analysis. Bivariate analysis was carried out to see the crude association between each independent variable and outcome variable. *P*-value < 0.05 and 95%CI for adjusted odds ratios (AOR) were used to declare the significance of the associations. The qualitative data were analyzed using thematic analysis.

**Result:**

The findings of this study showed that 76% of the households were food insecure. Being female-headed households [AOR: 2.82:CI(1.10, 7.24)], absence of formal education [(AOR: 9.75:CI (3.7, 11.31)], lack of engagement in non-farm farm activities [(AOR; 3.30: CI (1.86, 5.96)], absence of credit service [AOR:4.04; C I (2.11,7.73)], presence of dependent household members [AOR: 3.47;(2.91,6.34)], poorest wealth status [AOR;9.86:CI (3.72, 15.85)] were factors significantly associated with food insecurity of the households. Food insecure households employed different coping strategies with the respective level of food insecurity.

**Conclusion:**

The findings of this study indicated that household food insecurity was higher in the study area. Moreover, sex, educational status, wealth status of the households; engagement of households in off/non-farm farm activities, credit service, and active and inactive labor force were significantly associated with household food insecurity.

Food insecure households practice different coping strategies with respective food insecurity levels from the less severe strategy of eating inedible, fewer-quality foods to the most severe of migrating and begging for food.

Planning and exhaustively implementing sustainable food security programs should get due attention.

## Background

Food insecurity is a state in which people experienced limited or uncertain physical and economic access to safe, sufficient, and nutritious food to meet their dietary needs or food preferences for a productive, healthy, and active life [1]. It has two broad components: insufficient access to a nutritionally adequate and safe food supply at the household level, and inadequate utilization of these foods by household members [2].

Food insecurity continues to be a major development and public health problem across the globe, having adverse consequences for individuals, undermining people’s health, productivity, and often their survival [3, 4]. Households with insufficient access to food often face other challenges related to food insecurity including poor health and a decline in productivity that can often create a vicious circle where households are unable to produce enough food, even in good years, because they are battling chronic health issues and are unable to work to their full potential [5, 6].

Globally about 2 billion people (26.4%) percent of the world population in the world experience a moderate or severe level of food insecurity. Among these, 1.04 billion (52%) are found in Asia; 676 million (34%) are in Africa; and 188 million (9%) are in Latin America. The lack of regular access to nutritious and sufficient food that these people experience puts them at greater risk of malnutrition and poor health [4, 7].

Total food insecurity is much higher in Africa than in any other part of the world affecting more than half of the population with its varied effect within the regions; (Southern region 53.6%, Eastern region 62.7%, Western region of Africa 47.9%) [8, 9]. Ethiopia, East African country, is one of the poorest countries in which large portions, approximately 20.5%, of households are estimated to be food in secured implying that rural households are more food in secured than urban households [10].

Households that face the problem of food insecurity do not sit back in despair rather they employ different strategies to reduce, mitigate, and cope with the risks and shocks that affect them. However employed coping strategies were either severe, nutritionally-negative coping strategies or reversible (those that compromised nutritional health, fiscal stability or are illegal and are less reversible including low nutrient foods, skipping meals, consuming smaller portions, borrowing or hustling) or nutritionally-positive (those that increase availability of resources and nutritious foods) [11, 12]. As justified in similar studies copping strategies such as Selling household asset, dropping children out of schooling, eating seed stock and selling fire wood and/or charcoal are also common responses which could have a long term negative effect on the food security status of households in particular and the entire society in general [12, 13]. In Ethiopia, thus, depending on the magnitude of the duration and severity of food insecurity, coping mechanisms ranging from less to more extreme have been employed [14].

Southern Nation Nationalities and People Region (SNNPR) is a region that experienced high levels of food insecurity; nearly (55%), [1]. Accordingly, Kedida Gamela District; the study area is highly vulnerable to population pressure, frequently recurring drought, erratic rainfall pattern, flood, crop and animal disease as a result it experienced high level of food insecurity, child and maternal malnutrition (stunting, wasting and underweight), infection of malaria, starvation, dependency, drop out of education and migration.

Even though there are growing number of food insecurity studies conducted on both rural and urban set ups, the prevalence and associated factors varies even in the same region. In addition, there are few studies conducted that describe coping strategies employed by food in secured rural households; as a result the evidence available is critically scant in the country in general and in study area in particular. Therefore, this study aimed to assess households’ food insecurity, factors associated with households’ food insecurity and to explore copping strategies employed by food in secured households among rural households of Kedida Gamela District of Kembata-Tembaro, Zone in Southern Nation Nationalities and Peoples Region of Ethiopia.

## Materials and methods

### Study area

Kedida Gamela district is one of the seven administrative districts under Kembata-Tembaro Zone, South Nations Nationalities, and People Regional State of Ethiopia. The district is located 130 Km north west of Hawasa and 310 km south west of Addis Ababa, the capital city of Ethiopia. It has 11 administrative kebeles (the smallest administrative unit in Ethiopia), 2 health center, and 11 health posts. The district is located between the latitude of 7^0^ 11^′^N to 7^0^ 19^′^N and 37^0^50^′^ 30^“^E to 38^0^ 4^’^ 30^”^E longitude. The altitude of the district ranges from 1700 to 3028 m above sea level. The topography of the district includes highlands and plains. About 28% of the area is plains and 35% are high lands rough surfaces. Its area is divided into “dega”, 7% and “woyna dega”, 93%. According to 2017 central statistical agency population projection, the total population of the district is 124,338and average land holding is about 0.3–0.5 ha per household. Wheat, maize, root crop and “inset” are the most known perennial crops in the area.

### Study design and period

Community based cross-sectional study design complemented with qualitative inquiry was conducted from August up to November, 2020.

### Study population

Study participants for quantitative method were randomly selected rural households found in the kebeles of the district and for qualitative, food insecure households in randomly selected kebeles of the district. The study included all households with the respondents lived at least for 6 month in the study area. Household heads who were seriously sick and unable to respond for the questions during data collection period and mentally ill were excluded from the study.

### Sample size determination

The sample size was calculated using EPI-info version 7.2 Software for the estimation of sample size for single proportion. Taking the prevalence of household food insecurity 62% from similar study conducted in Mareko district in Guraghe zone Southern Ethiopia [10], 95% CI (1.96), assumptions of design effect 1.5, desired absolute precision of 5%, anticipated non–response 10%, the final sample size was 597**.**

### Sampling procedure

For the quantitative method, multi-stage sampling technique was used. Primary sampling units, 4 Kebeles were selected from total 11 rural kebeles in the District using simple random sampling technique (lottery). The secondary sampling units, the households in the selected Kebeles were selected by using simple random sampling technique. The calculated sample size 597 households were allocated proportional to population size for the selected 4 kebeles. All eligible households were identified based on list of households in household folder in respective kebeles and were interviewed until desired sample size is obtained.

For the qualitative study, an in-depth interview was conducted after receiving approval and getting individual consent to gather information from the respondents using In-depth Interview Guide (IDI) on household food insecurity coping strategies among purposively selected study participants (food in secured households). Totally sixteen food in secured households (13 MHHs and 3 FHHs) from previously selected four kebeles were included based on the folder number from the respective kebeles total households list and individual sampled household questionnaire ID number that also set on SPSS and food security status after quantitative analysis. The interview was conducted face to face and was involved one interview with one participant at a time within the participants’ choice in their home in order to obtain rich data on the way of safe environment. The researcher was engaged with participants posing questions in a neutral manner, listening attentively to participants’ responses and asking follow up and probes questions based on participants’ response. For each participant the interviews were conducted at the range of 40 to 60 minutes. The interviews were conducted by researcher in translating the English version open-ended interview guide to local language, Kembatigna. The interviews were tape recorded and short field notes were also used for non-verbal (facial, head nodding, etc.) expressions as a means of data collection through active interaction with researcher-participants; and transcribed verbatim by principal investigator in English,

### Data collection and measurement

For the quantitative study, a semi-structured interview administered data collection questionnaire was used to collect relevant data. The Household Food Insecurity Access Scale (HFIAS), a standard tool that has been developed by Food and Nutrition Technical Assistance (FANTA) and validated in several countries were adopted to assess household food security status. HFIAS deals with the occurrence and frequencies of occurrence of food insecurity, consisting of nine occurrence questions that represent a generally increasing level of severity of food insecurity (access), and nine “frequency-of-occurrence” questions that are asked as a follow-up to each occurrence question to determine how often the condition occurred [15]**.**

The questions focus on measuring the feeling of uncertainty or anxiety about food supply; insufficient quantity of food consumed; insufficient quality of food consumed; reported reduction of food intake or experience of hunger; and reported consequences of reduced food intake. These sets of questions are known to be used in several countries and appear to decide the food secure from the food insecure households across diverse cultural settings [16]**.**

A HFIAs score variable is computed for each household by summing up the codes for each frequency or occurrence question. Before summing the frequency of occurrence codes, the frequency of occurrence was coded “0” for all cases where the answer to the corresponding occurrence questions was “No”. The maximum score for a household was 27 (the household which responded to all nine frequencies of occurrence questions was often coded with response code of 3) the minimum score was 0 (the household responded “No” to all occurrence questions). The higher the score, the more food insecurity (access) the household experienced; the lower the score, the less food insecurity (access) a household experienced [15, 17].

For qualitative study, in-depth Interview Guide (IDI) was employed to explore coping strategies adapted by food in secured households. The guide mainly focused on participants’ opinions, believes, attitudes, behaviors, and perceptions regarding what they do when they don’t have enough food and money to buy food. The interview was conducted face to face and was involved one interview with one participant at a time within the participants’ choice in their home in order to obtain rich data on the way of safe environment. The researcher was engaged with participants posing questions in a neutral manner, listening attentively to participants’ responses and asking follow up and probes questions based on participants’ response. For each participant the interviews were conducted at the range of 40 to 60 minutes. The interviews were conducted by researcher in translating the English version open-ended interview guide to local language, Kembatigna. The interviews were tape recorded and short field notes were also used for non-verbal (facial, head nodding, etc.) expressions as a means of data collection through active interaction with researcher-participants; and transcribed verbatim by principal investigator in English.

### Data processing and analysis

For quantitative study, data were entered into Epi Data version 3.1 and exported to SPSS version 21 for analysis. Descriptive statistics like, mean, frequency were computed and presented by using text, tables and graph. Collinearity diagnostic test was conducted to check for collinearity between independent variables. The tolerance values for all of the independent variables were larger than 0.10. Model fitness was also checked by using Hosmer–Lemeshow Goodness-of-Fit Test (*p* = 0. 260). Binary logistic regression was undertaken to see association between dependent and independent variables. Variables having a *p*-value of < 0.25 in binary logistic regression were transferred to multivariable logistic regression. Odds ratios at 95% CI were computed to measure the strength of the association between the outcome and the explanatory variables. *P*-values less than 0.05 were considered as statistical significant in the multivariate analysis.

The wealth index was computed by PCA using data from DHS as a composite indicator of living standards based on asset ownership, source of water, housing characteristics, materials used for housing construction, presence of agricultural land and quantity of livestock. Variables were considered by coding 0 and 1 for the analysis. In confirmation of the assumptions of PCA, the Kaiser-Meyer-Olkin (KMO) measure of sampling adequacy and the significance of Bartlett’s test of sphericity were checked. During analysis, variables were dropped when their communality scores were less than 50%. Principal components having Eigen values greater than one and total variance that was above the recommended minimum value of 60% were identified. Wealth index values were calculated by summing up the scores for the three components. Ultimately, the four categories of rich, good, poor, and rich were generated by splitting the wealth index values into four equal classes.

For qualitative study, the information collected from in-depth interview with electronic tape and field notes were transcribed, translated, coded, synthesized and organized under thematic heading manually. First, the verbal data from the interviews were transcribed. The transcribed notes were then read repeatedly in order to become familiar with the breadth of the data. The process of searching for meanings and patterns of the important information was conducted simultaneously during the reading process. After that, the coding process was carried out manually. Each code that was identified, together with the relevant data extracts, were then kept in separate computer file. The codes identified were sorted into potential themes and the relevant coded data extracts were collected within the identified themes. The extracts of the data for each theme were reviewed according to their coherence. Therefore in-depth interview data were analyzed by thematic content analysis to created concepts from interview data and triangulated findings with quantitative evidences. The data were further evaluated and analyzed to determine its adequacy, credibility and usefulness to objectives of the study.

## Results

### Socio-demographic and socio-economic characteristics; and institutional factors

The study incorporated an overall sample of 582 households among the overall 597 households initially sampled, making the response rate 97.5%. Concerning the socio-demographic characteristics, the majority, 466 (81%), of households were mainly male-headed. The mean (+ SD) age of participants was 44.16 (+ 9.06) with minimum age of 25 and maximum age of 75 years. Nearly half (261, 49%) of the study participants were in the 18–35 years age group. Moreover, married households accounted for about 512(89%) of the study participants, and 262 (45%) of the households had a family size of 6–8 members. Less than half, (276, 47.4%) of the household heads had completed primary education. Principal Component Analysis (PCA) finding indicates that 116(19.9%), 113(19.4%), 121 (20.8%), 116(19.9%), and 116 (19.9%) have lowest, second, middle, fourth, and highest wealth status respectively among the participants (Table 1). Concerning the level of wealth status, 574(96.8%), 545(93.6%), 521(89.5%) had chair, own farm land, and Car/motorbike respectively (Table 2).

**Table 1:** Socio-demographic and socio-economic variables of participants in Kedida Gamela district in 2020

Variables (*n* = 582)	Categories	Frequencies	Percentages
Sex	Male	466	80.1
	Female	116	19.9
Age	18–35	261	44.8
	36–50	109	18.7
	51–64	200	34.4
	> 65	12	2.1
Marital status	Married	512	88
	Other wise	70	12
Family size	2–5	253	43.5
	6–8	262	45
	> 8	67	11.5
Main food sources	Purchase from market	266	45.7
	From own production	259	44.5
	Gift from others	57	9.8
Additional jobs	Off-farm activities	278	47.8
	No additional jobs	304	52.2
Educational status	No formal education	169	29
	Primary	276	47.4
	High school	117	20.1
	Above high school	20	3.4
Number of active	< 2 members	361	62
	3–4	180	31
Labor force	Above 4	41	7
Number of inactive labor force	< 2 members	157	27
	3–4	216	37.1
	Above 4	209	37.1
Monthly expense	<900ETB	149	25.6
	901–1400	148	25.4
	1401–2000	189	32.5
	>2000ETB	96	16.5
Wealth Status	Lowest	116	19.9
	Second	113	19.5
	Middle	121	20.8
	Fourth	116	19.9
	Highest	116	19.9
Credit	No	264	45.4
Service	Yes	318	54.6
Ag Input	No	153	26.3
	Yes	429	73.7
Ag Extension	No	138	23.7
	Yes	444	26.3
Access to saving	No	344	59.1
	Yes	238	40.9
Screened to PSNP	No	371	63.7
	Yes	211	36.3
Screened to Relief	No	371	69.4
	Yes	211	30.6

*Ag* Agricultural extension, *AI* Agricultural input

**Table 2:** Variables related to the level of wealth status/index in the study area

Variables	Categories	Frequencies	Percentages	Loading component
Own farm land	No	37	6.4	
	Yes	545	93.6	0.597
Oxen for farming	No	370	63.6	
	Yes	212	36.4	0.448
Having cows	No	236	40.6	0.746
	Yes	34	59.4	
Hen/chicken	No	349	60	0.721
	Yes	233	40	
Sheep/goats	No	272	46.7	0.498
	Yes	310	53.7	
Donkey	No	468	80.4	0.508
	Yes	114	19.6	
Beehive	No	414	88.3	0.455
	Yes	68	11.7	
Cart	No	478	82.1	0.541
	Yes	103	17.7	
Tape	No	446	71.5	0.727
	Yes	166	28.5	
Television	No	502	86.3	0.616
	Yes	80	13.7	
Watch	No	552	94.8	0.351
	Yes	30	5.2	
Mobile phone	No	402	69.1	0.602
	Yes	180	30.9	
Bed	No	91	15.6	0.562
	Yes	492	84.4	
Table	No	100	82.6	0.562
	Yes	100	17.4	
Chair	No	8	1.4	0.512
	Yes	574	98.6	
Car/motorbike	No	521	89.5	0.435
	Yes	61	10.5	
Water source	Pipe	300	51.5	0.709
	None-pipe	282	48.5	
Type of roof	Iron sheet	161	27.7	0.625
	Grass	421	72.3	
Type of house floor	Cemented	112	19.2	0.738
	Not cemented	470	80.8	

### Household food insecurity indicators and its status

Based on the evidence available household food insecurity access scale tool, households worried about not having enough food within the last 4 weeks were 515 (88%) among the study participants, and households that ate food really did not want were about 155(27%) in the study area. On the other hand, households ate smaller amount of food in a meal and fewer meals in a day, 312 (53.6%) and 262(45%) respectively among the study subjects. Furthermore, households going to sleep hungry at night were 82(14.1%) and no household had gone a whole day and night without food based on the current findings (Table 3).

**Table 3:** Household food insecurity access scale main variables in Kedida Gamela district 2020

Variables	Categories	Frequencies	Percentages
Worry about not having enough food	No	67	11.5
	Yes	515	88.5
Unable to eat preferred food	No	144	24.7
	Yes	438	75.3
Eat just a few kinds of food	No	181	31.1
	Yes	401	68.9
Eat food really do not want	No	427	73.4
	Yes	155	26.6
Eat smaller amounts in meal	No	270	46.4
	Yes	312	53.6
Eat fewer meals in a day	No	320	55.0
	Yes	262	45.0
No food of any kind in household	No	500	85.9
	Yes	82	14.1
Go to sleep hungry at night	No	554	95.2
	Yes	28	4.8
Go a whole day and night without food	No	582	100
	Yes	0	0

The HFIAS indicator categorizes households into four levels: food secure and mild, moderate, and severely food insecure. The findings of this study showed the degree (severity) of household food insecurity as 140 (24%), 109 (18.7%), 212 (36.40%), and 121 (20.8%) food secure, mild food insecure, moderate food insecure, and severe food insecure.

The study also further categorized the food security status into two categories: food secured and food insecure households. Thus, by merging mild, moderate, and severe levels 442 (76%) 95%CI (72.6, 79.4) of the study participants were food insecure and 140 (24%) 95%CI (20.6, 27.4) were food secure in the study area.

### Factors associated with household food insecurity

Bivariate and multivariable logistic regression analysis was done to analyze factors associated with household food insecurity. In the bivariate analysis setting, sex of the household head, marital status of the household head, educational status of the household head, monthly income of the household, wealth status of the household, additional job of the household head, main food source of the household, agricultural extension service, and active and inactive labor force in the household were significantly associated with household food insecurity in return candidates for multivariate analysis.

The multivariate logistic regression was done to identify predictors of household food insecurity. Variables with *P* < 0.25 in bivariate logistic regression and candidates for multivariable logistic regression were included in the final model. After controlling for the confounders, sex, educational status, wealth status, participation in additional jobs/off for farm activities, credit service, and active and inactive labor force were significantly associated with household food insecurity with a p-value < 0.05.

Female headed-households were 2.82 more likely to be food insecure than male-headed households [AOR: 2.82; CI (1.10, 7.24)]. Those households whose heads had no formal education were 9.7 times more food insecure than households headed by people with an above-high school level [(AOR: 9.75; CI (3.71, 11.31)].

Households with the poorest wealth status were 9.86 times more highly probable to be food insecure than households with the best/rich wealth status in the study area [AOR: 9.86; CI (3.72, 15.85)].Those households that haven’t used credit services were 4.04 times more food insecure as compared with those that had used credit services [AOR: 4.04; CI (2.11, 7.73)].

Households that did not participate in additional jobs/off for farm activities were 3.69 times more likely to be food insecure than their counterparts [AOR: 3.69; CI(2.03, 6.71)].

Those households that had more than four inactive labor force/dependent members were 3.47 times more likely to be food insecure than those have had less than two dependent members [AOR: 3.47;(2.91, 6.34)]. On the other hand, those households that had a less active labor force or independent household members were 3.57 times more likely to be food insecure as compared to those households that had more active labor force [AOR: 3.57;(1.16, 9.94)](Table 4).

**Table 4:** Factors associated with household food insecurity in Kedida Gamela district in 2020

Variables(*N* = 582)	Categories	HH food security status		COR (CI)	AOR (CI)
		Insecure (%)	Secure (%)		
Sex of HH	Male	337(73.3)	129(27.7)	1	1
	Female	105(90.5)	11(9.5)	3.65(1.90, 7.02)	2.82(1.10,7.24)*
Marital status	Married	376(73.4)	136(2.6)	1	1
	Ling with no partner	66(94.35)	4(5.7)	5.96(2.13,16.68)	2.18(0.61,7.76)
Main food sources	Purchase from market	206(77.4)	60(22.6)	0.12(0.03,0.52)	0.35(0.07,1.6)
	From own production	181(69.9)	78(30.1)	0.08(0.02,0.35)	0.19(0.04,0.9)
	Gift from others	55(96.5)	2(3.5)	1	1
Additional jobs	Off-farm activities	174(62.6)	104(37.4)	1	1
	No additional jobs	268(88.2)	36(11.8)	4.45(2.91,6.80)	3.69(2.03,6.71)*
Educational status of HH head	No formal education	199(91.7)	18(8.3)	12.16(5.60,26.4)	9.75(3.7,11.31)*
	Primary	194(78.5)	53(21.5)	4.04.(2.04, 7.92)	2.32(0.75,6.34
	High school	29(38.2)	47(61.8)	0.67(0.31,1.45)	0.28(0.43,1.22)
	Above high school	20(47.6)	22(52.4)	1	1
Number of actives	< 2 members	286(79.4)	75 (20.8)	3.63(1.87,7.04,)	3.57(1.16,9.94)*
	3–4	135(75.0)	45 (25.0)	2.85(1.42,5.74,)	2.05(0.68,4.14)
	Above 4	21(51.2)	20(48.8)	1	1
Number of inactive	< 2 members	96(61.1)	61(38.9)	1	1
	3–4	166(76.9)	50(23.1)	0.25(0.15,0.42)	1.54(0.78,3.20)
	Above 4	180(86.1)	29(13.)	0.53(0.32,0.88	3.47(2.91,6.34)*
Monthly expense	<900ETB	38(95.0)	2(5)	13.7(6.3,30.07)	0.65(0.47,1.90)
	901–1400	178(90.8)	18(9.2)	5.3(2.9,9.8)	0.95(0.33,2.70)
	1401–2000	213(68.1)	100(31.9)	1.7(1.1,2.8)	0.78(0.35,1.70)
	>2000ETB	13(39.4)	20(60.6)	1	1
Credit	No	180(68.2)	84(31.8)	2.18(1.48,3.21)	4.04(2.11,7.73)*
	Yes	262(82.4)	56(17.6)	1	1
Agricultural input	No	135(88.2)	18(11.8)	2.98(1.74,5.08)	1.97(0.63,6.19)
	Yes	307(71.6)	122(28.4)	1	1
Agricultural extension	No	124(89.9)	14(10.1)	3.50(1.94,6.33)	1.38(0.40,4.69)
	Yes	318(71.6)	126(28.4)	1	1
Wealth status	Lowest	110(98.8)	6(5.2)	17.71(7.21,23.3)	9.86(3.72,15.8)*
	Second	100(88.5)	13(11.5)	7.43(3.75,9.71)	4.23(0.96, 4.89)
	Middle	96(79.3)	25(20.7)	3.71(0.96,4.56)	1.78(0.84,2.96)
	Fourth	77(66.4)	39(33.6)	1.90(0.12,3.24)	0.91(0.09,1.98)
	Highest	59(50.9)	57(49.1)	1	

* Significant at *P*-value <0.05, *COR* Crude Odd Ratio, *AOR* Adjusted odd ratio, *CI* Confidence interval, *HH* Household, *ETB* Ethiopian Birr

### Coping strategies of food insecure households

#### Demographic and socioeconomic characteristics of the participants’

The in-depth interview was conducted among 16 food-insecure households in previously selected kebeles of the district. Among them, 2 (12.5%), 9 (56.25%), and 5 (31.25%) were mi1d, moderate and severely food insecure, respectively. The participants were aged between 35 and 62. Seven of them did not have any formal education and the rest of the nine participants learnt formal education at different levels. All of them are married households, and their total monthly income ranges from 900 to1400 ETB (Table 5).

**Table 5:** Demographic and socioeconomic characteristics of In-depth- Interview Participants in Kedida Gamela district in 2020

ID	Folder ID	SPSS ID	Kebele	Sex	Age	Educational Status	Food Insecurity status
1	Aze/158	009	AzeDebao	Male	46	No formal education	Moderate
2	Aze/523	065	AzeDebao	Male	50	No formal education	Mild
3	Aze/950	094	AzeDebao	Male	48	No formal education	Moderate
4	Aze/1460	121	AzeDebao	Male	35	High school	Severe
5	Abo/370	183	Abonsa	Male	50	Primary school	Severe
6	Abo/502	214	Abonsa	Male	47	Primary school	Severe
7	Abo/750	246	Abonsa	Male	49	Primary school	Moderate
8	Abo/800	274	Abonsa	Female	60	No formal education	Mild
9	Ker/101	302	Kerchicho	Male	50	Primary school	Moderate
10	Ker/200	334	Kerchicho	Male	50	Primary school	Moderate
11	Ker/956	371	Kerchicho	Male	46	Primary school	Moderate
12	Ker/1450	405	Kerchicho	Male	50	Primary school	Moderate
13	Ger/215	438	TezaGerba	Male	47	High school	Moderate
14	Ger/530	492	TezaGerba	Male	62	No formal education	Severe
15	Ger/880	530	TezaGerba	Female	55	No formal education	Severe
16	Ger/1154	557	TezaGerba	Female	48	No formal education	Moderate

*ID* Participant Identification number, *SPSS ID* Identification number on SPSS, *Folder ID* Identification number HH folder

The key informants noted various mechanisms that were employed at times of food shortage in the study area. Coping strategies such as reliance on less preferred and less expensive food, reducing the number and frequency of meals, borrowing food or relying on help from others, seeking wage labor, eating wild food and harvest immature crops, storing seed stock for the next season, selling productive and nonproductive assets, restricting consumption of adults to favor children to eat, borrowing money at high profit to buy food, sending household members elsewhere, partial and complete dependence on aid enforced to do unusual habits such as begging and temporal and permanent migration were employed in the study area.

The majority of key informants frequently stated that they were forced to seek unusual additional jobs such as wage, daily labor, or cash for work in order to save themselves and their families. One of the key informants stated the situation as;

*... We were forced to do daily labor work, shifting away from agriculture and working for food in relatives or neighbors in order to get a daily meal. Not only were we, but our children had also skipped school and performed the same task to earn money for food (male participant, 35).*

The participants also noted that;

*… We were forced to depend on less preferred and less expensive foods because the preferable foods were run out and we had no money to buy food. As a result, we ate what we had to sustain our life or for survival (male participant aged 49).*

The study participants noted that they rely on less preferred or inedible and less expensive food aimed at maintaining quantity rather than quality of food.

*… we had to depend on less preferred and less expensive foods because the preferable foods were run out and we had no money to buy foods. As a result, we ate what we had got to sustain our life or for survival (male participant aged 46).*

The participants explained skipping meals and limiting portion size as coping methods when they face insufficient access to food.

*… we mostly worry about whether we are going to eat well tonight rather than the quality of food. We often do not have the choice of what we will eat, but rather eat when food is available. One or two meals a day is common (male participant aged 50).*

Other participant also said;

*... not only do we skip meals and limit the amount and portion size of food we eat, but we also limit our consumption as adults to feed our children. Therefore, the question of food security is not pertinent, but mostly the issue is survival (male participant aged 47).*

The study participants also noted that they have experienced un-habitual actions as coping mechanisms that are considered as shameful activities in the community during the normal season.

*… we borrowed money to pay back at high interest, in addition to borrowing food when the food shortage became severe and we passed that bad condition. (female participant aged 50).*

Participants that were at serious food shortage noted that;

*… I and my family rely on help from others mostly; sometimes we also buy food on credit (male participant aged 48).*

Similarly other participants also remind the situation as;

*… We do not have anything to eat as a result of the rent of land, but it was not sufficient to cover our living costs and we sold the three that were left to construct a house. (female participant aged 55).*

Few key informants stated that they are forced to take harsh actions such as dropping children from school, sending them elsewhere for work and begging, going without food during the day and/or night, seasonal and permanent migration, and begging. As shown in table 6, coping strategies reported by key informants were grouped under three themes based on the level of severity of coping strategies. Insurance strategies are sometimes called less severe coping strategies or first stage coping strategies; crises or erosive coping strategies are sometimes called second stage coping strategies; and distress or failed strategies are known as stage three coping strategies. The socioeconomic status and the notes of KIIs showed that food insecure households use one or more coping strategies with their respective levels of food insecurity.

**Table 6:** Themes developed for coping strategies adapted by food in secured HHs in the district, 2020

First Theme	Second Theme	Third Theme
Insurance strategies	Crisis Strategies	Distress Strategies
Rely on less preferred and less expensive foods. Inter-household transfers of food &/or money Taking out of loans from relatives Reduction in number and frequencies of meal in a day Reduces expenses on daily necessities Seeking for wage or daily labor to rise income: ✓ Cash for work, ✓ Moving from agriculture and become daily laborer or ✓ Involve on off-farm and pity trade activities	Taking loan or borrow money at high interest rate to buy food Borrow and / or buy food on credit and rely on others help Gathering wild foods or harvest immature/ unripe food/ crops Eat seed for the next planting season Sending household members children) to eat at friends’ or relative’s Limiting the portion size at meal time restrict the number of meals eaten Skipping meals or restrict consumption by adults to feed children Going the whole days with little food Depend on relief assistance Rent/contract farm land Sell of non-productive assets Temporary/Seasonal migration search of wage employment	Eating too less amount and less nutritious or un edible meals Sell of all live stocks and productive assets/equipment Sell or mortgage of land Children dropping out of school and sending them elsewhere to work and beg. Go the entire day (the whole day and night) without eating Permanent Migration Complete dependence on aid Engaged in begging to get food and resources

The findings of the current study showed that food insecurity occurrence indicators in HFIAS were noted by key informants during the qualitative study one more time, depending on the food insecurity level of the participants. This can be justified as the types of coping strategies used by food insecure households as indicators of food insecurity. This showed that the result of the qualitative finding explains the quantitative result. In addition to this, the key informant interview report and socioeconomic data of the key informants showed that the types of coping strategies used as markers of the level of food insecurity (Table 6).

## Discussion

The findings of this study indicate that 76% of the study participant households were food insecure in the study area. Gender, sex, monthly income, educational status, and wealth status of the household; participation in additional jobs or off-farm activities; having credit or borrowing money in high profit for purchasing food; and the number of inactive labor force or family members were the key predictors. On the other hand, based on the qualitative evidence on copping strategies of household food insecurity, households practice eating inedible food staffs, eat smaller/fewer foods, and less frequently. Moreover, households cop by borrowing money and getting food help, working for food, eating on debt and using reserves frequently, and households’ members in the current setting migrate and beg for food less frequently as per the current study findings.The prevalence of household food insecurity reported in this study was lower in comparison with the studies done in the North Eastern Peninsular of Malaysia, Sekyere-Aframplain of Ghana and Sidama district, Southern Ethiopia, which were reported as 83.9,79 and 82.3% respectively [18–20]; and also higher than the findings from the Edo state in Nigeria, Tharanka in Kenya, Damot Gale district in Ethiopia, which were reported as 52.7, 47 and 71.6% respectively [7, 9, 21]. However, the findings of this study were comparable to the findings reported by studies conducted in the South Delhi district of India (77.2%); East Badewacho district (75.8%) and Kindodidaye district (71.6%) in Ethiopia [1, 3, 20]. The difference in the findings might be due to variation in study settings and data collection seasons.

The relatively higher prevalence of household food insecurity in this study might be due to the data collection period, which was a season with food access shortages due to the pandemic COVID 19 across the globe in general and in the study area specifically. This might have overestimated the magnitude of the problem. Thus, seasonal data with repeated surveys may give better evidence.

Furthermore, as previously stated, food insecure households employ multiple coping strategies to combat existing household food insecurity, which increases the quantitative evidence validity indicating the extent of household food insecurity in the study setting. Based on the varied informant interviews, the qualitative evidence on the copping strategies of household food insecurity indicates that households practice eating inedible food items and eat smaller/fewer foods most frequently. Moreover, households cope by getting food help, working for food, eating on debt, and using reserves frequently. This study’s findings are somewhat consistent with those of a study conducted in Ethiopia’s Amaro and Mareko districts [10, 22]. On the other hand, household members in the current setting migrate and beg for food frequently, as per the current study findings. Quantitative evidence also shows that food insecure households borrow money at high profit, rely on emergency relief, and screen productive safety net programs. In general, the qualitative findings on copping strategies indicate that food insecure households adapt different copping strategies from simple to severe with their respective food insecurity levels. Female-headed households were 3.5 more likely to be food insecure than male-headed households. The reason for this finding might be land cultivation and crop production activities that improve food security, which are culturally covered mainly by males rather than females in the study area. The finding was in line with a similar study done in the west Abaya district in Southern and Shalla district in Oromia region in Ethiopia [23, 24].

Households who gained 1500–2500 per month were 5.7 times more likely to be food insecure than households that gained 3500 per month. The findings of this study indicate monthly income as one of the key predictors of household food insecurity. Based on the findings, households that gain relatively lower per month were more likely to be food insecure than households that gain a better income per month in the study area. Research done in South Africa also boosted the validity of evidence by reporting that households with higher monthly incomes were less likely to suffer from household food insecurity than households with lower incomes [25]. The evidence is consistent with the finding reported from Edo state of Nigeria, West Abaya and Shalla districts in Ethiopia which reported as monthly income as predictor for the household food insecurity in the similar direction [7, 23, 24]. The finding reported in this section could be explained by households that have better access to food than those that do not. Besides, household members’ efforts to works for a wage to increase income to cope with household food insecurity in the study area based on the qualitative evidence. That indicates having a better income matters more than having a diet and validates the predictor’s soundness for the outcome variable.

The educational status of the household head has had statistically significant link with the state of household food insecurity. That is, illiterate households were more likely to be food insecure in the study area in comparison to households headed with better educated households.

The finding is consistent with similar studies done on rural households’ food security status and coping strategies in Edo State, Nigeria [7]; and the studies done in Humbo, Amaro, Damot Gale,Mareko, and East Badewacho [1, 20, 22, 23]. The evidence is straightforward that trained household heads have better access to food than untrained groups of households. This can be justified as educational attainment by the household heads could lead to better awareness of possible advantages of modernizing agriculture and also improve the chance to diversify the household’s income source, which in turn would enhance the households’ food supply.

Households with the Poorest wealth status were 5.2 times more likely to be food insecure than households with best/rich wealth status in the study area. That is; poorer households were 5.2 times more likely to be food insecure than wealthier households. When we increase household food security, better wealth status has a statistically significant association.. Even though there were no exact indications, most variables used in this study as wealth were significantly associated with the dependent variable; for instance, land size as a variable was indicated. Thus, the current finding is comparable with a study done on rural households’ food security status and coping strategies in Edo State, Nigeria, that reported as land size had a significant association with the outcome variable [7]. The possible reason for this finding should be still the wealthier households have the better capability to access food than poorer households as per this study findings.

Households engaged in off-farm activities like pity trade in the study area were better food secure or less likely to be food insecure than their counterparts. On the other hand, households who don’t participate in off-farm income generating activities were expected to face a food deficit because their farm income was found to be insufficient enough to cover households’ food needs. The findings of this study are in line with the study conducted in the Asosa, Mareko and Kindodidaye districts in Ethiopia [10, 20, 26]. Households engaged in off-farm activities like petty trades in the study area were found to be more food secure than their counterparts.

The findings of the current study revealed that food in secured households employed different copping strategies and decisions to mitigate the effect of not having enough food to meet the household’s needs. The use of these copping strategies by food insecure households was mainly based on the magnitude and severity of food insecurity, ranging from less severe to very severe.

Insurance or first stage copping strategies are less severe; second stage or crisis strategies are severe; and very severe copping strategies are distress or failed copping strategies [27]. The current study also found that less severe and severe coping strategies are used more frequently than very severe coping strategies among food insecure households, initially shifting from protection of economic assets to disposal assets and finally to destitution, indicating that the severity of the situation influences the type of coping strategies used, which is consistent with the findings of Hadley and Crooks [4]. Less severe or insurance strategies are often used by mild and moderate food insecure households to minimize the risk of food insecurity and to improve income and reduce asset depletion on the other hand. Severe and/or very severe copping strategies that are mainly characterized by consumption of less diversified foods and foods low in nutrient content may affect the nutritional status and the health condition of children as well as the well-being of the overall family. In addition to this severe and/or very severe coping strategy such as sending children elsewhere, migration and begging are highly shamed and odd habits, unusual in society. Copping strategies such as borrowing food on credit or money at a high interest rate, selling productive and non-productive assets, and consuming seeds for the next plantation season may have an even greater impact on household livelihood. This is partially in lined with the finding of Maxwell and studies conducted in Ethiopia [4, 5, 28, 29].

## Conclusion

The prevalence of food insecurity in the study area was high. Being female headed households, illiterate educational status, households with poorest wealth status, Households with more dependent labor force, households having credit to buy food, and engagement of households in off-farm activities were factors associated with households’ food insecurity.

Food insecure households practice different copping strategies with their respective food insecurity levels from less severe copping strategies to the most severe ones. As a result, the most commonly used less severe and/or severe copping strategies were eating inedible, lower quality and less nutritious food, eating smaller/fewer foods, borrowing money, seeking wage labor, and cash for work. On the other hand, they get food help, work for food, eat on debt, and use reserves frequently, and household members migrate and beg for food less frequently used copping severe&/or very severe copping strategies in the study area. Raising awareness, family planning, and improving household food security strategies that protect against the use of severe and most severe copping strategies should be prioritized.

## Data Availability

The data that support the findings of this study are available from corresponding author upon reasonable request.

## Abbreviations

AOR: Adjusted Odds Ratio
CI: Confidence interval
CSI: Copping Strategy Index
EDHS: Ethiopian Demographic Health Survey
ETB: Ethiopian Birr
FANTA: Food and Nutrition Technical Assistance
FAO: Food and Agriculture Organization
FHH: Female Headed Households
HFIAS: House Hold Food Insecurity Access Scale
LFS: Low Food-insecure
MHH: Male Headed Households
PSNP: Productive Safety net Program
VLFS: Very Low Food –insecure
SNNPR: Southern Nation Nationality People Regional
SPSS: Statistical Package for Social Science
UNICEF: United Nations Children Fund
VIF: Variance Inflation
